# Vitreous Humor Versus Peripheral Blood for Accurate Estimation of Antemortem Alcohol Levels: A Systematic Review of Postmortem Human Studies

**DOI:** 10.7759/cureus.99092

**Published:** 2025-12-13

**Authors:** Abdulkreem Al-Juhani, Rodan Desoky, Naif Aljohani, Omar O Aljohani, Renad A Abdulkareem, Reyof N AlQumaizy, Hatoon Hakeem, Abdulelah Alasmari, Muhannad A Badghaish

**Affiliations:** 1 Forensic Medicine, Forensic Medicine Center, Jeddah, SAU; 2 Surgery, Faculty of Medicine, King Abdulaziz University, Jeddah, SAU; 3 College of Medicine, Alfaisal University, Riyadh, SAU; 4 Forensic Medicine, Forensic Medicine Center, Riyadh, SAU; 5 Forensic Medicine, Ministry of Health, Riyadh, SAU; 6 Medicine and Surgery, Almaarefa University, Diriyah, SAU; 7 General Practice, Ministry of Health, Riyadh, SAU; 8 Forensic Medicine, Forensic Medicine Center, Al Madinah, SAU; 9 Forensic Medicine, Ministry of Health, Jeddah, SAU

**Keywords:** blood alcohol concentration, decomposition, etg, ethanol, ets, forensic toxicology, hs‑gc, postmortem interval, quadas‑2, vitreous humor

## Abstract

Estimating antemortem blood alcohol concentration (BAC) after death is complicated by postmortem artifacts (endogenous ethanol formation, contamination, redistribution). Vitreous humor is anatomically protected and may resist these artifacts, offering a more stable matrix. The detection of ethyl glucuronide (EtG) and ethyl sulfate (EtS) can further distinguish true antemortem intake from postmortem production. Our objective is to evaluate the diagnostic reliability of vitreous ethanol concentration (VAC) as a surrogate for antemortem BAC, and to define contexts where vitreous testing adds forensic value. This review of postmortem human studies compared VAC with BAC, while adhering to the Preferred Reporting Items for Systematic Reviews and Meta-Analyses (PRISMA) 2020 guidelines for systematic reviews. We searched PubMed, Scopus, Web of Science, and Google Scholar from July 2010 to July 2025, screened in duplicate, extracted data using a standardized form, and appraised study quality with a revised Quality Assessment of Diagnostic Accuracy Studies-2 (QUADAS-2) tool for forensic accuracy. Given methodological and clinical heterogeneity (analytical techniques, postmortem interval (PMI), decomposition), we performed a structured narrative synthesis. Nine studies (Europe and Asia) met our inclusion criteria. Most used headspace gas chromatography with flame ionization detection (HS‑GC/FID) for ethanol; several incorporated liquid chromatography-tandem mass spectrometry (LC-MS/MS) for EtG/EtS. VAC and BAC were strongly correlated (r ≈ 0.89-0.97) under standardized sampling and shorter PMIs. Vitreous matrices showed greater resilience in cases with decomposition, long PMI, or suspected microbial ethanol formation. Studies measuring EtG/EtS indicated that combined ethanol-metabolite interpretation improves specificity for antemortem drinking and reduces false positives. Despite high correlation, individual BAC predictions from VAC had wide confidence/prediction intervals, underscoring the need for contextual, multi‑parameter interpretation. QUADAS‑2 suggested a low risk of bias in most domains across included studies. Meta‑analysis was not feasible due to heterogeneity. In conclusion, vitreous ethanol is a forensically robust adjunct or alternative to peripheral blood when BAC is unavailable or compromised, particularly with decomposition or suspected postmortem ethanol formation. Interpretation should pair VAC with EtG/EtS, femoral BAC (when available), PMI, and scene/autopsy data. Standardizing analytical protocols and validating calibrated prediction models across PMI and decomposition strata are priorities to reduce case‑level uncertainty.

## Introduction and background

Forensic toxicology necessitates precise antemortem blood alcohol concentration (BAC) estimations. Nonetheless, postmortem artifacts such as endogenous ethanol generation due to microbial activity, gastrointestinal contamination, and redistribution effects may undermine blood-based investigations [1,2]. These artifacts are challenging to utilize in decomposed remains or extended postmortem durations, resulting in inaccurate conclusions regarding alcohol consumption before death [3,4]. The vitreous humor, a transparent gel located in the posterior chamber of the eye, serves as a promising alternative matrix owing to its anatomical isolation, bacterial resistance, and stability after death [5,6]. When peripheral blood is compromised due to decomposition or trauma, this fluid remains largely protected from contamination and postmortem redistribution.

Vitreous fluid retains ethanol concentration more effectively, even in cadavers with prolonged postmortem periods. Direct ethanol metabolites such as ethyl glucuronide (EtG) and ethyl sulfate (EtS), which are only produced in vivo, have been demonstrated to validate antemortem alcohol consumption in vitreous humor [6-8]. The integrated evaluation of ethanol and its metabolites enhances the forensic reliability of toxicological data and diminishes false positives associated with decomposition. Owing to diminished ocular clearance kinetics, vitreous alcohol concentrations (VACs) may surpass BAC during the post-absorptive phase [2,9,10].

Thus, alcohol concentration discrepancies between matrices indicate physiological changes that must be considered during interpretation. This relationship has been examined in forensics to create vitreous BAC prediction algorithms. These models have vast confidence intervals, necessitating contextual interpretation despite their capabilities. Despite this variation, numerous studies have reported significant correlations (r > 0.9) between BAC and VAC, showing excellent dependability under standardized conditions [5,10,11]. These findings justify utilizing vitreous fluid instead of blood alcohol in many forensic circumstances, particularly when blood is unavailable, compromised, or when postmortem alcohol production is suspected. Vitreous examination helps, and we found that vitreous ethanol concentrations match femoral blood measurements under controlled conditions [11,12]. We observed that vitreous ethanol can aid, although determining BAC from it is problematic, especially in specific cases [13]. Indeed, forensic toxicologists must use vitreous data, scene evidence, postmortem findings, and EtG and EtS biomarkers. This extensive investigation compares postmortem vitreous humor to peripheral blood ethanol. We aim to evaluate vitreous ethanol as a surrogate marker for antemortem alcohol levels when blood measurement is hampered by decomposition, contamination, or other postmortem alterations. VAC-BAC linkages' therapeutic and forensic effects and toxicological methods' vitreous tests are discussed in the article.

## Review

Methodology

**Figure 1:**
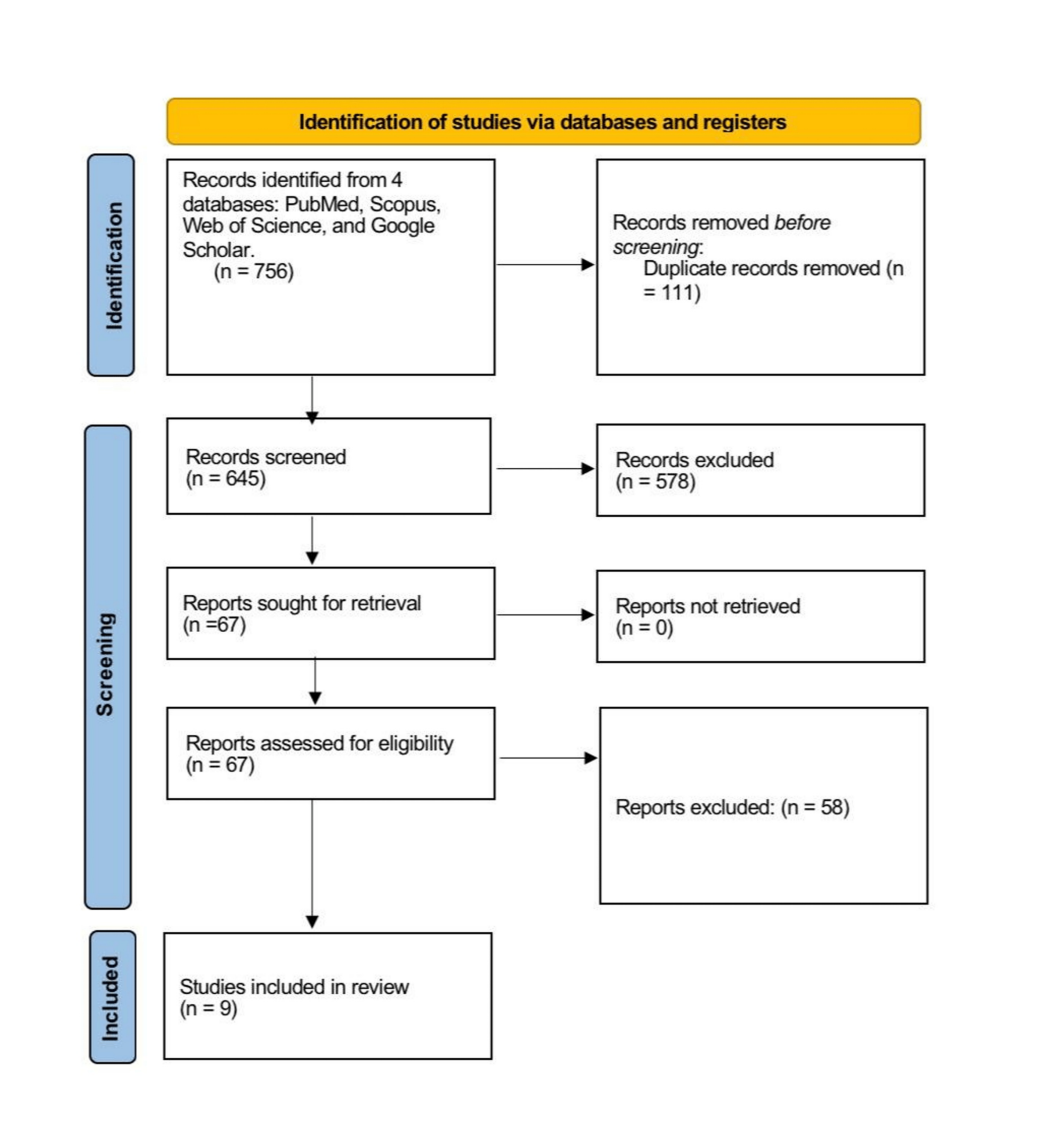
PRISMA flow diagram PRISMA: Preferred Reporting Items for Systematic Reviews and Meta-Analyses

This systematic review adhered to the Preferred Reporting Items for Systematic Reviews and Meta-Analyses (PRISMA) guidelines (Figure 1). The study compared the ethanol concentration in vitreous humor (VAC) to the peripheral BAC in deceased forensic autopsy subjects to assess diagnostic reliability. The review analyzed postmortem human studies on vitreous fluid as a biological matrix for assessing antemortem alcohol levels, particularly in instances of decomposition or suspected ethanol intoxication. The Population, Intervention, Comparator, and Outcome (PICO) framework structured eligibility criteria.

The group comprised deceased forensic patients necessitating a BAC estimate. The intervention assessed ethanol concentrations in the vitreous humor, while the comparator evaluated it in peripheral blood. The primary discovery was vitreous ethanol's capacity to accurately indicate antemortem alcohol consumption, considering postmortem delay, degradation, and microbial ethanol synthesis. We exclusively analyzed distinctive human observational studies, including retrospective reviews, autopsy case series, and cross-sectional analyses.

Excluded research involved animal models, experimental techniques, or lacked a VAC-BAC comparison. PubMed, Scopus, Web of Science, and Google Scholar were examined from July 2010 to July 2025. The search terms comprised "vitreous humor," "ethanol," "alcohol," "postmortem," "blood alcohol concentration," "forensic toxicology," and "ethanol correlation." No temporal restrictions were used; however, only papers published in the English language were considered.

We conducted a comprehensive examination of the reference lists of all included papers to ensure completeness. Grey literature and unpublished research were omitted. EndNote X9 (Clarivate, London, UK) preserved all citations, whereas Rayyan (Qatar Computing Research Institute, Doha, Qatar) conducted the screening. Two independent reviewers evaluated the relevance of titles and abstracts following the removal of duplicates. Complete texts of possibly relevant studies were obtained and evaluated based on inclusion and exclusion criteria. Consensus or third-party consultation resolved disputes regarding research inclusion. The informal evaluation demonstrated exceptional inter-rater reliability. The selection process is illustrated in the PRISMA flow diagram. Two reviewers independently extracted data utilizing a standardized form. Data were retrieved on study design, publication year, country of origin, sample size, cause of death, analytical method (e.g., gas chromatography), postmortem interval (PMI), decomposition or putrefactive changes, VAC and BAC ethanol concentrations, and statistical measures of correlation or agreement.

A revised Quality Assessment of Diagnostic Accuracy Studies-2 (QUADAS-2) tool for forensic accuracy studies evaluated bias and methodological rigor of the incorporated research. The risk of bias in patient selection, index test (VAC), reference standard (BAC), and sample collection schedule was evaluated. Studies with high bias across many areas were dismissed. All nine papers in the final evaluation satisfied the inclusion criteria. A quantitative meta-analysis proved unfeasible due to clinical and methodological heterogeneity, especially regarding analytical techniques, PMIs, and decomposition processes.

A structured narrative synthesis examined the dependability of VAC and BAC across studies. Analysis of vitreous humor is valuable in forensic science, particularly when peripheral blood is compromised or when postmortem ethanol is generated.

Results

Nine postmortem human studies comparing peripheral blood ethanol concentrations (BAC) and vitreous humor ethanol concentrations (VAC) were included in this systematic review to evaluate the accuracy of antemortem alcohol level estimation.

The comprehensive features of the included studies are compiled in Table 1, which includes information on authors, publication years, countries of origin, sample sizes, study strategies, and analytical techniques used. Due to the geographical diversity of the studies, which covered forensic environments in Germany, Romania, Italy, Poland, Sweden, Norway, Austria, and Thailand, the findings had broad worldwide applicability. In terms of methodology, the majority of research used flame ionization detection (FID) in conjunction with headspace gas chromatography (HS-GC) to measure the amount of ethanol. The analytical robustness of the included investigations was further validated by a number of studies that used sophisticated liquid chromatography-mass spectrometry (LC-MS/MS) techniques to detect ethanol metabolites, specifically EtG and EtS.

**Table 1:** Study characteristics (chronologically ordered) Key details of each included study, including authors, publication year, country, sample size, study design, and analytical methods used for ethanol measurement. EtS: ethyl sulfate; EtG: ethyl glucuronide; HS-GC: headspace gas chromatography; LC-MS: liquid chromatography-mass spectrometry; FID: flame ionization detection; VH: vitreous humor

Study (Year)	Country	Sample Size	Design	Analytical Method(s)
Thierauf et al. (2011) [4]	Germany	Not reported (autopsy cases with ethanol use)	Cross-sectional autopsy case series	HS-GC for ethanol; LC-MS/MS for EtG and EtS measurement.
Ioan et al. (2015) [5]	Romania	202 autopsy cases	Retrospective forensic case series	Cordebard micro-distillation oxidation method (colorimetric) for ethanol (official Romanian method).
Vezzoli et al. (2015) [6]	Italy	63 autopsy cases	Retrospective observational study	HS-GC-FID for ethanol and putrefactive alcohols; LC-MS/MS for EtG.
Szeremeta et al. (2018) [7]	Poland	62 autopsy cases	Retrospective cross-sectional study	HS-GC with FID for ethanol in blood and vitreous.
Thelander et al. (2020) [8]	Sweden	Not reported (autopsy cases with biofluids analyzed)	Retrospective analytical study	HS-GC (headspace) on dual capillary columns for ethanol (n-propanol and t-butanol internal standards).
Savini et al. (2020) [9]	Italy	31 autopsy cases	Prospective analytical study (method validation and case analysis)	HS-GC-FID (validated 6-min run) for ethanol in blood and vitreous.
Neumann et al. (2021) [10]	Germany/Austria	117 autopsy cases	Retrospective cross-sectional study	HS-GC for ethanol in blood, urine, VH; LC-MS/MS for EtG in all fluids.
Oshaug/Høiseth et al. (2022) [12]	Norway/Sweden	Large datasets - 2504 cases (EtS dataset) and 8001 cases (putrefactive alcohols dataset)	Retrospective registry study	HS-GC for ethanol; LC-MS/MS for EtS in blood; GC for putrefactive alcohols (e.g., 1-propanol).
Boonyoung and Sirijanchune (2025) [11]	Thailand	108 autopsy cases (traffic fatalities)	Retrospective case series (traffic deaths)	HS-GC-FID for ethanol in femoral blood and vitreous humor (hospital forensic lab).

A unique thematic synthesis of VAC and BAC results in several forensic circumstances is presented in Table 2. Strong correlations between VAC and BAC were consistently shown in all experiments (correlation coefficients ranging from r = 0.89 to r = 0.97), confirming the vitreous humor's validity as a stand-in for antemortem blood alcohol contents. Importantly, vitreous humor demonstrated exceptional stability and resilience to postmortem changes, which makes it especially useful in forensic situations involving considerable decomposition, lengthy PMIs, or probable endogenous ethanol generation. The usefulness of EtG and EtS in differentiating between genuine antemortem alcohol intake and postmortem ethanol production was highlighted by Thierauf et al., Vezzoli et al., and Neumann et al. [4,6,10], among other papers that particularly addressed ethanol metabolites. By greatly improving forensic interpretations and lowering the possibility of false-positive results in decayed bodies, this distinction proved crucial. Notably, research by Boonyoung and Sirijanchune (2025) [11] and Thelander et al. (2020) [8] showed that even with high average correlations, individual BAC predictions based solely on VAC should be handled carefully because of the large prediction intervals, highlighting the need for additional interpretative context.

**Table 2:** Thematic comparison of vitreous versus blood alcohol findings Studies are grouped according to their primary focus: (a) correlation or prediction between vitreous alcohol concentration (VAC) and blood alcohol concentration (BAC), and (b) use of ethanol metabolites (ethyl glucuronide (EtG) and ethyl sulfate (EtS)) and decomposition markers to aid interpretation of VAC-BAC results. VH: vitreous humor; PMI: postmortem interval; NaF: sodium fluoride; PA: putrefactive alcohol; LC-MS: liquid chromatography-mass spectrometry; COD: cause of death

Theme	Study (Year)	VAC Versus BAC Ethanol Findings	Correlation (r)	Postmortem Interval (PMI)	Decomposition/Putrefaction	EtG/EtS Marker Results	Alcohol-Related COD?
VAC-BAC Correlation Studies	Ioan et al. (2015) [5]	No systematic VAC-BAC difference; mean VAC/BAC ratio ~1.1. Identified a formula to estimate BAC at death from autopsy, BAC, and PMI.	0.89 (VAC-BAC)	9-96 h (average ~24 h) ; longer PMI modestly lowered BAC (p=0.011).	Not specific (included some putrefied cases; method applicable even with trauma or embalming).	No direct EtG/EtS data (focus on ethanol only).	Mixed causes (not only intoxication; various forensic cases).
	Szeremeta et al. (2018) [7]	No significant difference between VH and blood ethanol levels; vitreous is a reliable alternative to blood.	0.96 (Spearman)	Unreported (routine autopsies 2012-2016; presumably within days).	Not specifically examined (likely excluded extreme decomposition).	No EtG/EtS reported (ethanol only).	Mixed (various autopsies, some ethanol-positive but not necessarily COD).
	Savini et al. (2020) [9]	VAC slightly higher than BAC in 74% of cases (VAC > BAC in 23 of 31, BAC > VAC in 8); no statistically significant mean difference. VAC/BAC ratio ~1.07.	R^2 = 0.9227 (r≈0.96)	Samples analyzed within ~5 days postmortem (stored 4°C with NaF).	Not a major factor (study included mostly intact bodies; vitreous is used when the body is putrefied or damaged, but decomposition impact was not explicitly analyzed).	No EtG/EtS measured (ethanol only).	Mixed (cases with ethanol present; not exclusively alcohol-overdose deaths).
	Thelander et al. (2020) [8]	Very high VAC-BAC correlation; mean femoral BAC ≈ vitreous ethanol (1.15 g/L mean in VH vs 1.12 g/L in blood) when averaged. However, a large 95% prediction interval (~±0.42 g/L for vitreous) means individual VAC-to-BAC estimates are uncertain.	0.97 (VH) (all correlations p<0.001)	Not stated (medicolegal autopsies; likely within days postmortem).	Some cases showed evidence of postmortem ethanol (analysis included alternative fluids to flag endogenous production). Recommended using multiple specimens to verify ethanol origin.	No direct metabolites measured; relied on presence of “higher alcohols” (e.g., n-propanol) to infer fermentation.	Mixed (alcohol involved in deaths to varying degrees; not limited to intoxication fatalities).
	Boonyoung and Sirijanchune (2025) [11]	BAC and VAC were highly consistent (mean BAC 182 mg/dL vs VAC 192 mg/dL). VAC slightly exceeds BAC on average (mean B/V ratio = 0.95, i.e., VAC ~5% higher). Cases >12 h PMI tended to have VAC ≥ BAC.	0.92 (Pearson)	All autopsies within 24 h of death. Longer PMIs (~12 h) are associated with vitreous > blood ethanol (likely post-absorptive phase).	Minimal decomposition (fresh bodies ≤1 day). Study notes VAC is more reliable if >12 h postmortem (less subject to early postmortem changes).	No (ethanol only).	No (cause of death was trauma from accidents, not acute alcohol poisoning; alcohol impairment was a contributing factor).
EtG/EtS and Decomposition Studies	Thierauf et al. (2011) [4]	VAC ethanol is used as a robust matrix to confirm pre-death drinking. Noted that VAC can exceed BAC in the post-absorptive phase. Significant BAC-VAC correlations reported (r>0.9 in prior studies).	(Not reported here; prior literature r≈0.94-0.97)	Unreported (cases included both fresh and some decomposed remains).	Emphasizes vitreous stability in decomposition: vitreous is less prone to postmortem ethanol generation.	Demonstrated EtG and EtS detection in vitreous as definitive proof of antemortem ethanol ingestion. Mean EtG in VH ~2.1 mg/L vs 4.3 mg/L in blood (urine ~62.8 mg/L). EtS was detected consistently (stable even in putrefied samples).	Mixed (focus on marker evidence of drinking, irrespective of COD - e.g., includes cases where alcohol use preceded death, not necessarily alcohol toxicity).
	Vezzoli et al. (2015) [6]	Identified “neo-formation” of ethanol: 13 cases had BAC >0.05 g/L but no EtG in blood/VH, indicating purely postmortem ethanol (8 of these had acetaldehyde and n-propanol present - putrefaction markers). In contrast, 14 cases with BAC >0.46 g/L showed EtG >0.01 mg/L in blood and VH, confirming antemortem alcohol use.	N/A (focus was on presence/absence of markers rather than correlation)	Unreported (mix of recent and moderately decomposed bodies, 2003-2011).	Explicitly evaluated: presence of n-propanol and acetaldehyde in blood/VH flagged decomposition-produced ethanol. 8/13 “EtG-negative” ethanol cases showed such putrefactive alcohols.	EtG measured by LC-MS/MS in blood and VH: cases with true antemortem intake had EtG in both matrices (up to ~3 mg/L in blood, ~2.9 mg/L in VH). EtG absence (with ethanol present) signaled postmortem artifact.	Mixed (included cases of suspected postmortem alcohol and real intoxication; aim was to distinguish them, not restricted to alcohol-overdose deaths).
	Neumann et al. (2021) [10]	In 39/117 cases, ethanol was detected in blood, urine, and VH; all three matrices correlated strongly (p<0.00001). One case had 0.11 g/L in blood but 0.00 in VH - indicating likely postmortem blood ethanol (confirmed by absence of EtG). Overall, vitreous ethanol levels mirrored blood levels in genuine antemortem drinking cases.	0.97 (estimated from significance; strong VH-blood correlation in ethanol-positive subset)	Unreported (autopsies in a forensic setting; varied PMIs).	Considered: cases where ethanol in blood lacked corresponding VH ethanol were linked to decomposition (endogenous production). EtG was especially useful in such cases.	EtG found in 62 cases (incl. some where BAC was 0) - indicating prior alcohol use even when ethanol had metabolized or evaporated. More cases were EtG-positive than ethanol-positive, due to longer metabolite persistence. EtG absence in ethanol-positive cases flagged possible postmortem ethanol (no antemortem intake).	Mixed (not limited to intoxication fatalities; sample covers various causes, using EtG to confirm drinking history).
	Oshaug et al. (2022) [12]	Examined large cohorts for postmortem ethanol artifacts. 15.3% of BAC-positive cases had no EtS in blood - implying postmortem ethanol formation. In EtS-negative cases, ethanol concentrations tended to be low: in vitreous, 77.8% were <0.20 g/kg (but a few blood samples (>4%) were ≥1.0 g/kg despite no EtS). Urine and VH often showed ethanol even when blood was EtS-negative (9.4% and 7.4% of cases, respectively). This indicates occasional significant fermentation of ethanol even in VH.	N/A (statistical frequency study; no direct BAC-VAC correlation reported)	Not explicitly (large registry data; broadly includes immediate to long PMI cases). Many EtS-negative cases were from decomposed bodies.	Yes - primary focus. Putrefactive alcohols (PAs like 1-propanol) appeared in 24.4% of cases. Central (chest cavity) blood had significantly more false-positive ethanol cases (EtS-, PA+) than peripheral blood. Ethanol ratios (VH/BAC, urine/BAC) were much lower in cases with PM ethanol (EtS- or PA+), though with high variability.	EtS (a purely antemortem metabolite) was tested: its absence despite measurable ethanol strongly signaled postmortem origin. Recommended using EtS and PA analysis to improve the accuracy of interpretation.	Mixed (all were cases with positive BAC regardless of COD; the goal was to identify false positives in any context, not focusing on intoxication deaths specifically).

Using the QUADAS-2 assessment technique [14], Table 3 provides a thorough analysis of methodological quality and risk of bias. Low risks of bias were found in all included studies in key areas, such as patient selection, index test performance (VAC analysis), use of a trustworthy reference standard (BAC analysis), and sample collection flow and timing. This research's uniform methodological rigor provides assurance on the reliability and validity of the combined data.

**Table 3:** Methodological quality assessment using QUADAS-2 Risk of bias and applicability concerns for each study were evaluated across the following domains: patient selection, index test (vitreous alcohol concentration (VAC) measurement), reference standard (blood alcohol concentration (BAC) measurement), flow and timing, overall risk of bias, and applicability of results. QUADAS-2: Quality Assessment of Diagnostic Accuracy Studies-2; EtS: ethyl sulfate; EtG: ethyl glucuronide; HS-GC: headspace gas chromatography; LC-MS: liquid chromatography-mass spectrometry; FID: flame ionization detection; VH: vitreous humor; PMI: postmortem interval

Study (Year)	Patient Selection Bias	Index Test (VAC) Bias	Reference (BAC) Bias	Flow and Timing	Overall Risk of Bias	Applicability Concerns
Thierauf et al. (2011) [4]	Low - Included relevant autopsy cases of ethanol use (appropriate spectrum)	Low - Blinded lab analysis of VH EtG/EtS and ethanol (validated methods)	Low - BAC measured by standard GC (independent gold standard)	Low - Paired blood and VH samples from each case; minimal delay between sampling matrices	Low overall bias	Low concerns - Applicable to forensic cases with possible alcohol use (markers generalizable)
Ioan et al. (2015) [5]	Low - Consecutive autopsy cases (broad inclusion)	Low - Used official method for VH ethanol (Cordebard oxidation; adequately validated)	Low - Reference BAC measured with same robust method	Unclear - Not all cases had vitreous collected (85/202 cases in VAC-BAC analysis) , though sampling times were identical for those tested	Low (no major bias; minor missing-data issue)	Low concerns - Reflective of typical autopsy scenarios (findings applicable despite older method)
Vezzoli et al. (2015) [6]	Low - Autopsy cases spanning various conditions (appropriate mix)	Low - LC-MS/MS for VH EtG (high specificity); HS-GC for ethanol (objective measurement)	Low - Femoral blood ethanol by HS-GC (gold standard); EtG by LC-MS/MS	Low - All included cases had both VH and blood tested; simultaneous sampling at autopsy	Low overall bias	Low concerns - Directly applicable to determining antemortem alcohol via metabolites in postmortem cases
Szeremeta et al. (2018) [7]	Low - Relevant sample of forensic cases (with blood and VH available)	Low - Standard GC-FID used for VH ethanol (laboratory-based, no observer bias)	Low - Reference BAC by same GC-FID method (accepted standard)	Low - Both matrices analyzed for every included case; short interval (same autopsy)	Low overall bias	Low concerns - Supports general use of VH as alternative matrix in routine forensic practice
Thelander et al. (2020) [8]	Low - Broad selection of medicolegal cases (multiple fluid types analyzed)	Low - HS-GC with dual columns for VH ethanol (very reliable technique)	Low - BAC by duplicate HS-GC analysis (high accuracy)	Low - All alternative specimens and blood taken at autopsy; complete paired data for regression	Low overall bias	Low concerns - High applicability to forensic analysis; highlights uncertainty in individual predictions but reinforces generalizability
Savini et al. (2020) [9]	Low - Autopsy cases with range of BAC levels (appropriate for validation)	Low - GC-FID method validated for VH ethanol (precise and blinded)	Low - BAC by GC-FID (standard); lab personnel presumably blinded between matrices	Low - All cases had paired VH/BAC results; samples handled identically (analysis within 5 days)	Low overall bias	Low concerns - Applicable to fresh cases and method development; results generalizable to similar forensic settings
Neumann et al. (2021) [10]	Low - Included 117 consecutive autopsy cases with toxicology (representative sample)	Low - Automated HS-GC for VH ethanol; LC-MS/MS for EtG (objective, quantitative)	Low - Reference BAC by HS-GC (gold standard); EtG in blood by LC-MS/MS	Low - All cases had blood, VH, urine tested for both ethanol and EtG; single time-point per case	Low overall bias	Low concerns - High applicability to forensic toxicology (evaluating ethanol origin in autopsy cases)
Oshaug et al. (2022) [12	Low - Very large datasets from routine casework (robust population)	Low - Index “test” was analysis of VH/urine ethanol and markers (objective lab data)	Low - Reference standard: peripheral blood ethanol with confirmatory markers (high-quality data)	Low - Comprehensive data; some cases lacked EtS or PA analysis (separate cohorts) but sample sizes very large; timing differences inherent but mitigated by size	Low overall bias	Low concerns - Broadly applicable to postmortem cases, especially in detecting fermentation artifacts (high external validity)
Boonyoung and Sirijanchune (2025) [11]	Low - All eligible traffic fatality autopsies included (clearly defined group)	Low - Standard GC analysis of VH ethanol (routine forensic lab procedure)	Low - Reference BAC by same standard GC method (reliable benchmark)	Low - Paired blood and VH obtained within 24h postmortem for all; no losses or delays	Low overall bias	Low concerns - Focused on trauma cases ≤24h PMI, but findings (VAC-BAC correlation) remain applicable to other short-PMI forensic cases

This systematic review's novelty and importance come from its comprehensive and well-organized thematic synthesis, which clearly explains the situations in which vitreous humor ethanol analysis provides more forensic value than a conventional peripheral blood sample. The benefits of vitreous humor, especially its stability in situations with advanced decomposition or postmortem artifact development, are clearly highlighted by the comprehensive review. Additionally, the vitreous humor's forensic usefulness is greatly strengthened by the explicit inclusion and evaluation of ethanol metabolites (EtG and EtS), reaffirming its function as a vital supplement or substitute for peripheral blood in postmortem toxicological investigations. This thorough method gives forensic professionals precise instructions on how to choose the right biological matrices, enhancing interpretative precision in a variety of forensic situations.

Discussion

This systematic review analyzes and compares the efficacy of peripheral blood ethanol concentrations (BAC) versus vitreous humor ethanol concentrations (VAC) as a reliable indicator of antemortem alcohol intake. Across the nine included studies, VAC consistently shows a strong correlation with BAC, with correlation coefficients ranging from 0.89 to 0.97. In general, vitreous humor proved to be more stable and less affected by postmortem changes than blood [4-12]. The anatomical and physiological isolation of the vitreous humor with low susceptibility to bacterial contamination and putrefactive changes, as well as the ease of sampling, are the key contributing factors to the stability and reliability of VAC in reflecting the antemortem alcohol levels [13]. Studies by Thierauf et al. (2011) [4] and Vezzoli et al. (2015) [6] analyzed the integration of ethanol metabolites, specifically EtG and EtS, further enhancing the reliability of VAC in the interpretation of postmortem alcohol findings. Despite the collective agreement on the effectiveness and reliability of VAC in reflecting the antemortem blood ethanol levels, some studies cautioned that individual BAC prediction solely from VAC can show wide prediction intervals [8,11].

The evidence concluded from the synthesis - the strong VAC-BAC agreement - aligns with the internationally established protocols that recommend vitreous humor analysis as a crucial adjunct in forensic investigations [2,3,14-16]. Our results strengthen the case for vitreous humor analysis by providing data-driven evidence that supports and complements the existing literature. Recent reviews further support the role of VAC in distinguishing true antemortem ingestion of alcohol from postmortem alcohol formation, particularly when combined with EtG and EtS, which is consistent with several of the findings in our included studies [6,11-13,17-19]. However, our results also align with the limitations discussed by Thelander et al. (2020) [8], which indicate that relying solely on VAC for the precise calculation of BAC should be avoided, given the substantial uncertainty associated with linear regression models that may lead to misinterpretation of true antemortem ethanol levels. Prolonged PMIs, decomposition, and individual biological variability further limit predictive reliability. Hence, VAC should be regarded as a supportive measure rather than a conclusive determinant of intoxication levels in medico-legal cases [10].

Across the nine included studies, the quality of evidence was high, with an overall low risk of bias, and the use of validated analytical techniques, such as HS-GC and LC-MS/MS metabolites, supports the reliability and methodological rigor. The QUADAS-2 (Table 3) assessment showed that all nine studies’ risk of bias was rated low in selection, test methods, reference, and timing. Although the settings across the studies were different, there was a consistent strong correlation between the VAC and BAC levels (r = 0.89-0.97). Altogether, the low risk of bias, robustness of the methodology, and reproducible correlations across heterogeneous forensic contexts provide strong confidence in the utilization of vitreous humor as a reliable adjuvant in antemortem blood alcohol levels estimation.

In forensic toxicology, finding the antemortem BAC is fundamental in various investigations [1]. The postmortem blood alcohol measurements are often compromised by factors such as contamination, decomposition, postmortem redistribution, and endogenous postmortem alcohol production [2,3]. With the vitreous humor's unique anatomical and physiological properties, it provides an isolated, well-preserved environment for the antemortem alcohol concentrations. Because of the vitreous humor’s biological nature and its avascularity [19], postmortem changes interfere with VAC only minimally, making it a reliable indicator for BAC when peripheral blood is compromised [6,7,11,12].

The included studies had several methodological strengths; however, some limitations should be acknowledged. Sample sizes in many studies were modest, restricting the generalizability of their findings [5,8,9,11,12]. Methodological variations existed across the studies, from analysis of the data to inappropriate reporting of key case-level variables, including exact PMI, decomposition status, cause of death, or storage conditions [6-12]. Moreover, variability in the sample selection, time since death, and biochemical stability of ethanol concentrations in different body fluids may have introduced bias into the observed correlations, especially with individual results [20-22]. Additionally, a number of limitations in our study warrant careful consideration when interpreting its findings. Since there is a limited number of high-quality studies comparing VAC-BAC, only nine studies were included, restricting the strength of conclusions. Our study only included papers in English, which may introduce a language bias, and the reliance on published peer-reviewed studies carries a possible publication bias, particularly towards papers with strong VAC-BAC correlation.

Future research should aim to overcome these gaps through large-scale, multicentric collaborations applying standardized VAC-BAC correlation, PMI-stratification designs, as well as integrating ethanol metabolites (EtG, EtS) and putrefactive markers with VAC analysis [15,16,23]. Such efforts would enhance VAC’s operational value and expand its usage across diverse postmortem scenarios.

## Conclusions

Across nine postmortem human studies, VAC correlated strongly with femoral/peripheral BAC (typically r ≈ 0.89-0.97) and demonstrated greater stability in the face of decomposition, contamination, and postmortem redistribution. When blood is unavailable, compromised, or susceptible to endogenous ethanol formation, vitreous sampling provides a reliable surrogate for antemortem alcohol assessment, especially when interpreted alongside EtG/EtS and putrefactive markers. However, while population‑level associations are robust, individual BAC back‑calculations from VAC carry wide prediction intervals and should be contextualized with PMI, scene findings, autopsy data, and microbial indicators. Methodological quality was generally good by QUADAS‑2, but heterogeneity in analytical protocols and reporting precluded meta‑analysis. Best practice is paired femoral blood-vitreous sampling with HS‑GC for ethanol and LC‑MS/MS for direct metabolites, standardized PMI documentation, and explicit handling of decomposition. Future work should develop calibrated prediction models stratified by PMI/decomposition stage and validate combined ethanol-metabolite algorithms to narrow uncertainty in case‑level estimation.
